# Supplementary material for: Metal-organic framework template-guided electrochemical lithography on substrates for SERS sensing applications
Source: Nat Commun. 2023 Sep 20;14:5860. doi: 10.1038/s41467-023-41563-5 (PMC10511444; doi:10.1038/s41467-023-41563-5)
Supplement: Supplementary file 1 — Supporting Information [file 41467_2023_41563_MOESM1_ESM.pdf]

Supplementary Information

Metal-organic framework template-guided electrochemical lithography on substrates for SERS sensing applications

Lu et al

Supplementary Figures

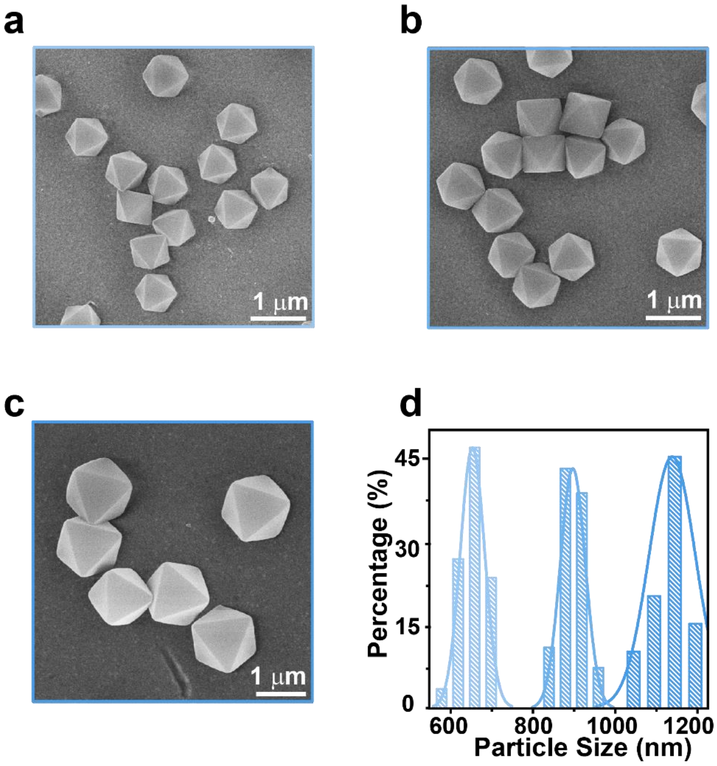

Supplementary Figure 1. SEM images and the corresponding size distribution of UiO-66 octahedra prepared at different temperatures. (**a** to **c**) 125 °C, 120 °C and 115 °C, respectively. (**d**) The corresponding size distribution. Source data are provided as a Source Data file.

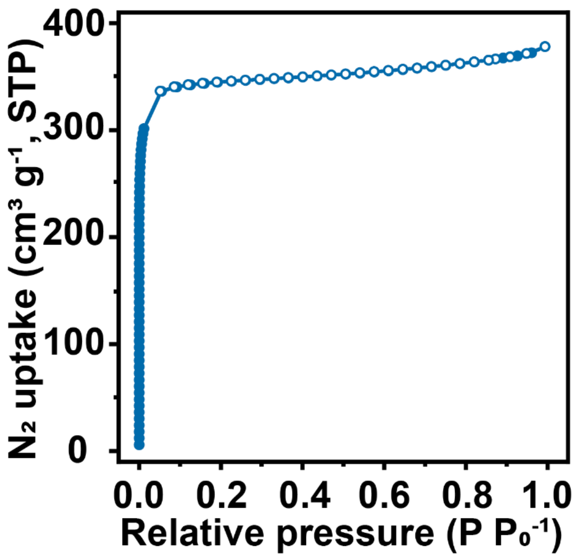

Supplementary Figure 2. N_2 adsorption isotherms of UiO-66 octahedra at 77 K. The solid and empty symbols represent the adsorption and the desorption process, respectively. Source data are provided as a Source Data file.

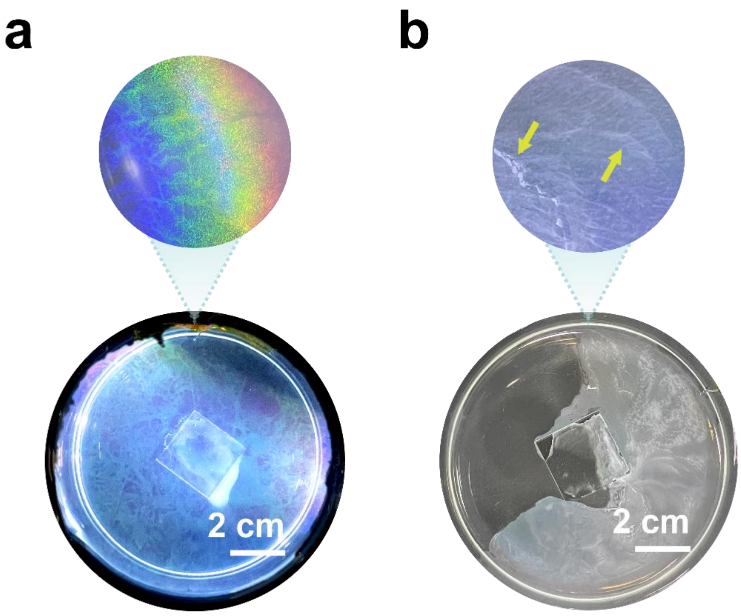

Supplementary Figure 3. Monolayer UiO-66 octahedron template floating on the water surface without and with defects. (**a**) The assembled monolayer UiO-66 octahedron template without defects. (**b**) The assembled thin film of UiO-66 octahedra with defects. Yellow arrows indicate the regions formed by multiple layers of UiO-66 octahedra.

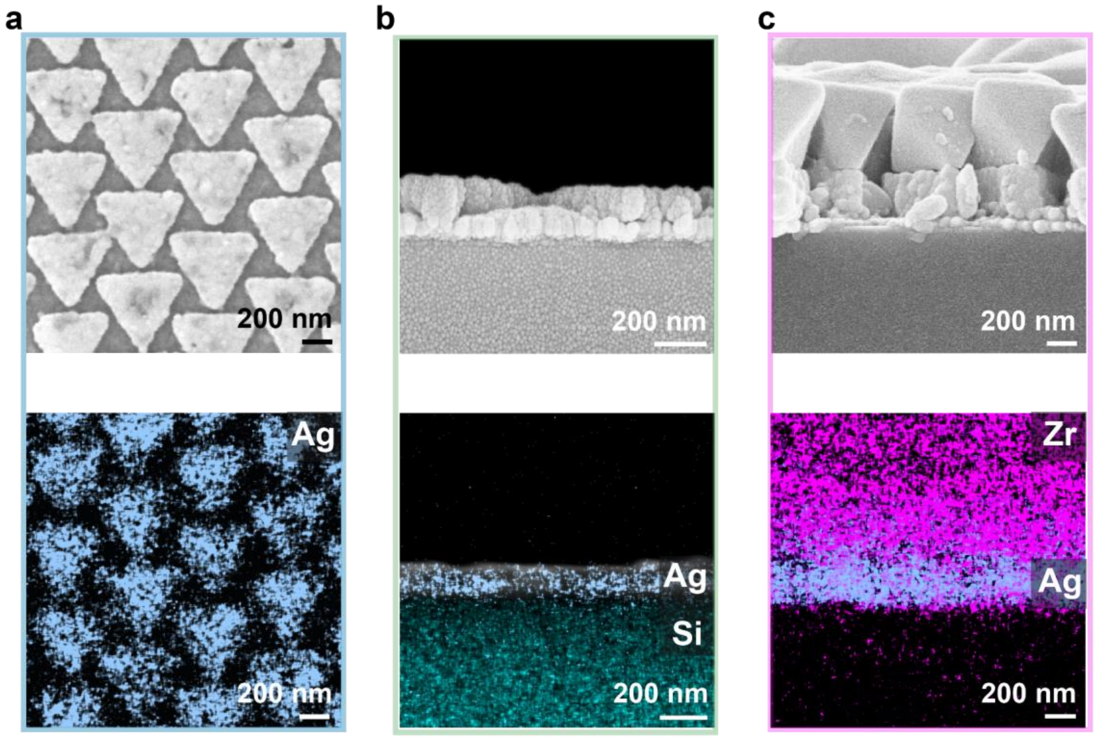

Supplementary Figure 4. Element mapping of Ag nanotriangle arrays. (**a**) Top view of Ag nanotriangle array electrodeposited for 15 s. (**b**) Side view of the Ag nanotriangle array electrodeposited for 15 s. (**c**) Side view before removing the monolayer UiO-66 octahedron template. The electrodeposition time was 300 s. The electrolyte composed of 300 mM $AgNO_3$ and 14 mM SDS and the electrodeposition voltage was 1.2 V. The same electrolyte was used for all of the following experiments, unless otherwise specified.

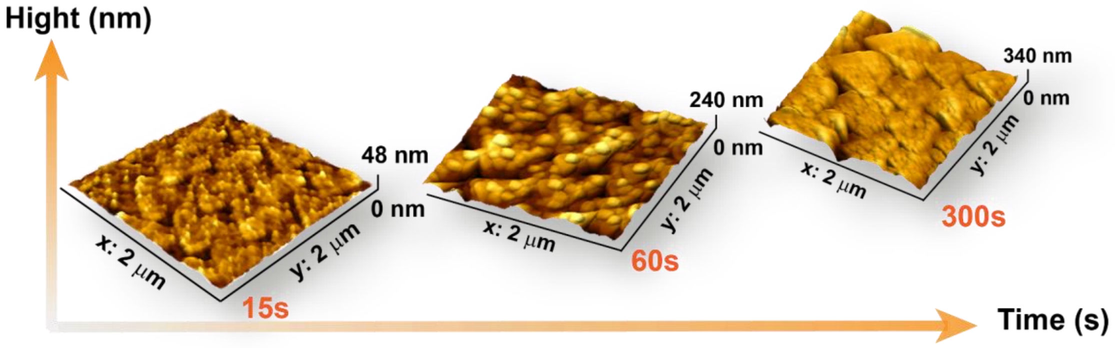

Supplementary Figure 5. AFM images of Ag nanotriangle arrays electrodeposited for different times. The electrodeposition time was increased from 15 s to 60 s and further to 300 s.

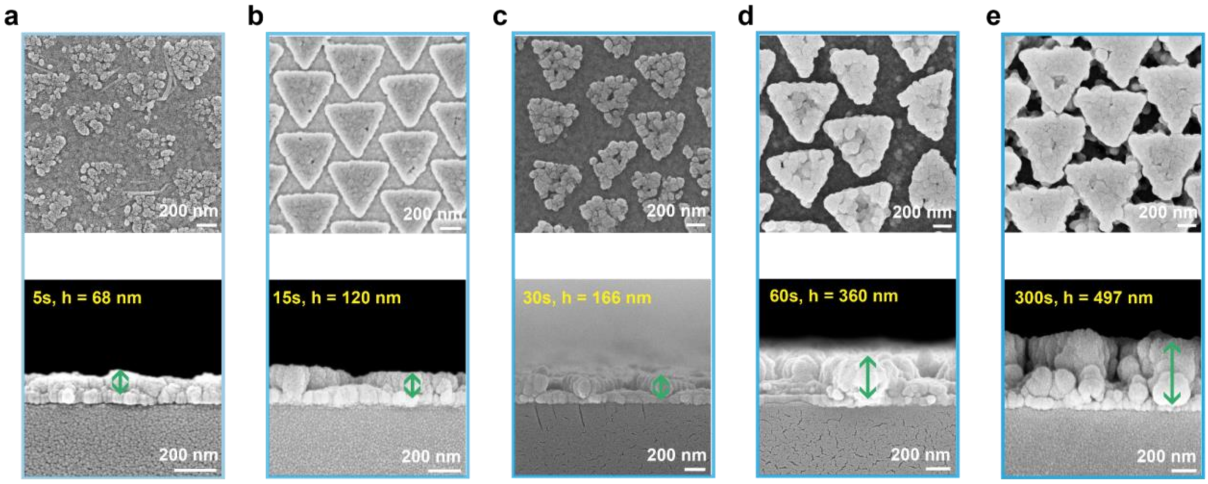

Supplementary Figure 6. Morphology and thickness evolution of the Ag nanotriangle arrays as the electrodeposition proceeded. (a to **e)** The electrodeposition time was 5 s, 15 s, 30 s, 60 s, and 300 s, respectively. The green arrows in the bottom panels of **a** to **e** showed the height growth of the Ag nanotriangles as the electrodeposition time was prolonged.

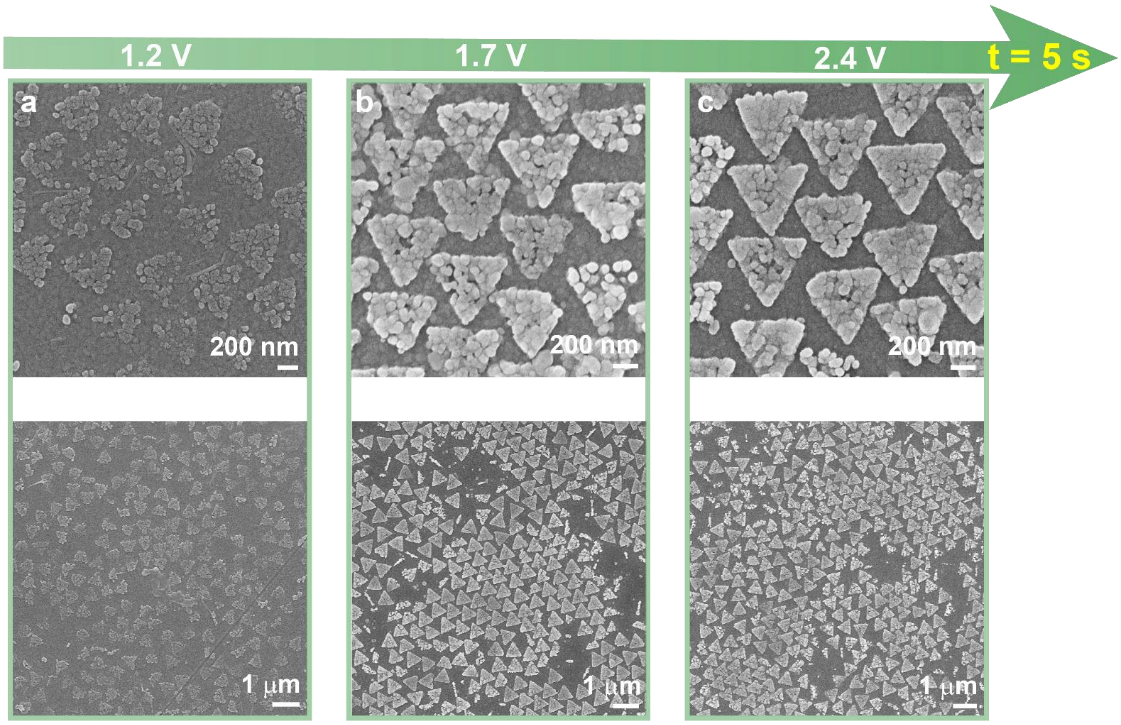

Supplementary Figure 7. SEM images of Ag nanotriangle arrays electrodeposited in a two-electrode system under different voltages for 5 s. (**a** to **c**) The electrodeposition voltage was 1.2 V, 1.7 V, and 2.4 V, respectively. The top and the bottom panels in **a** to **c** corresponded to the small- and large-area observations, respectively.

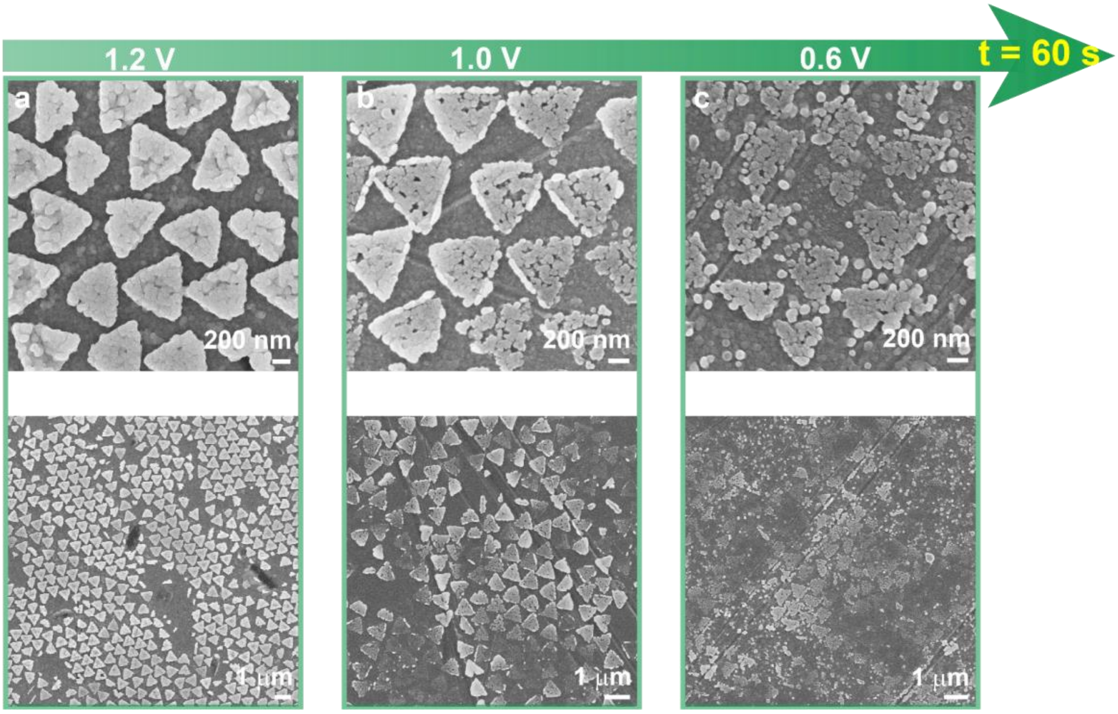

Supplementary Figure 8. SEM images of Ag nanotriangle arrays electrodeposited in a two-electrode system under different voltages for 60 s. (**a** to **c**) The electrodeposition voltage was 1.2 V, 1.0 V, and 0.4 V, respectively. The top and the bottom panels in **a** to **c** corresponded to the small- and large-area observations, respectively.

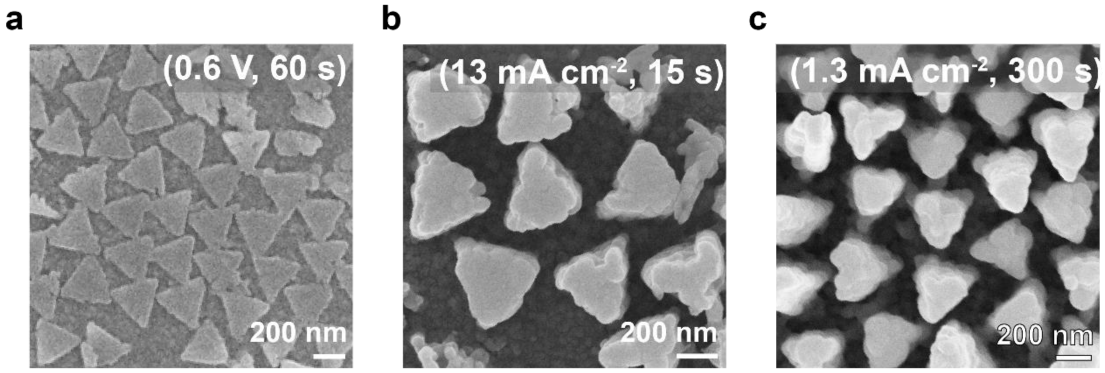

Supplementary Figure 9. SEM images of Ag nanotriangle arrays electrodeposited in a three-electrode system using Hg/Hg_2SO_4 as a reference electrode. (**a**) Applying a constant voltage of 0.6 V for 60 s. (**b**) Applying a constant current density of 13 mA cm^{-2} for 15 s. (**c**) Applying a constant current of 1.3 mA cm^{-2} for 300 s.

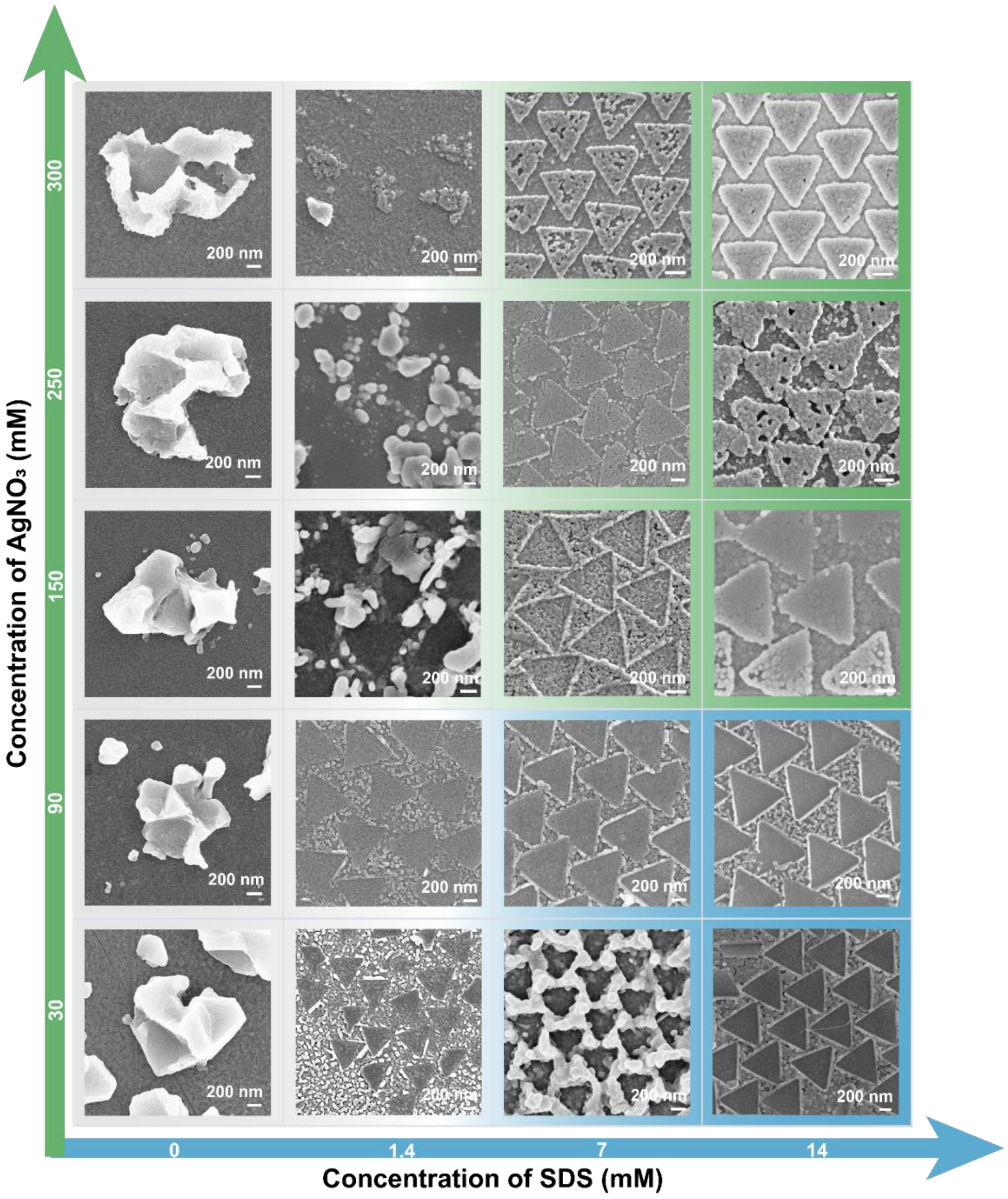

Supplementary Figure 10. SEM images of Ag nanostructures obtained in the electrolyte composed of different concentrations of $AgNO_3$ and SDS. The electrodeposition was performed at 1.2 V for 15 s except for (C_{AgNO3}, C_{SDS}) = (30 mM, 7 mM), where the electrodeposition time was 60 s.

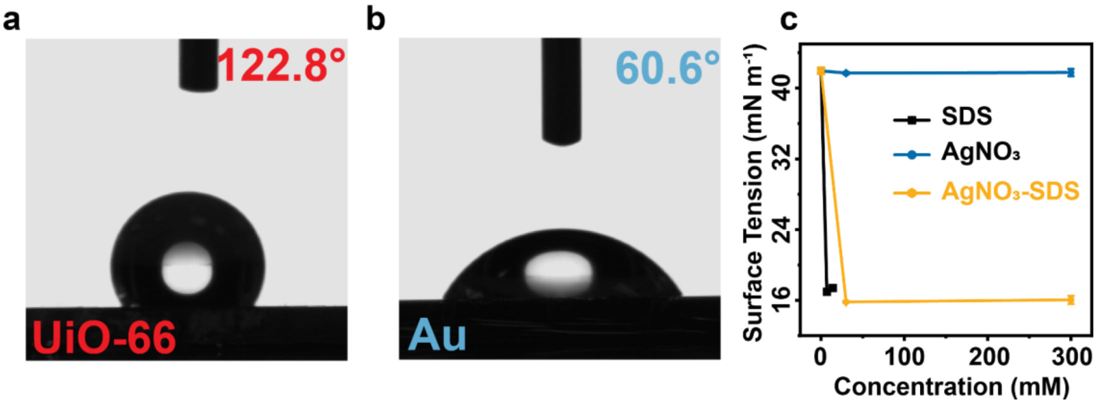

Supplementary Figure 11. Wettability characterization. (**a, b**) The water contact angle of the monolayer UiO-66 octahedron template and the bare gold substrate. (**c**) Surface tension of different solutions. Black curve: solutions composed of different concentrations of SDS. Error bars represent the standard deviation of three measurements of a sample. Blue curve: solutions composed of different concentrations of $AgNO_3$. Orange curve: solutions composed of 14 mM SDS and different amounts of $AgNO_3$. Source data are provided as a Source Data file.

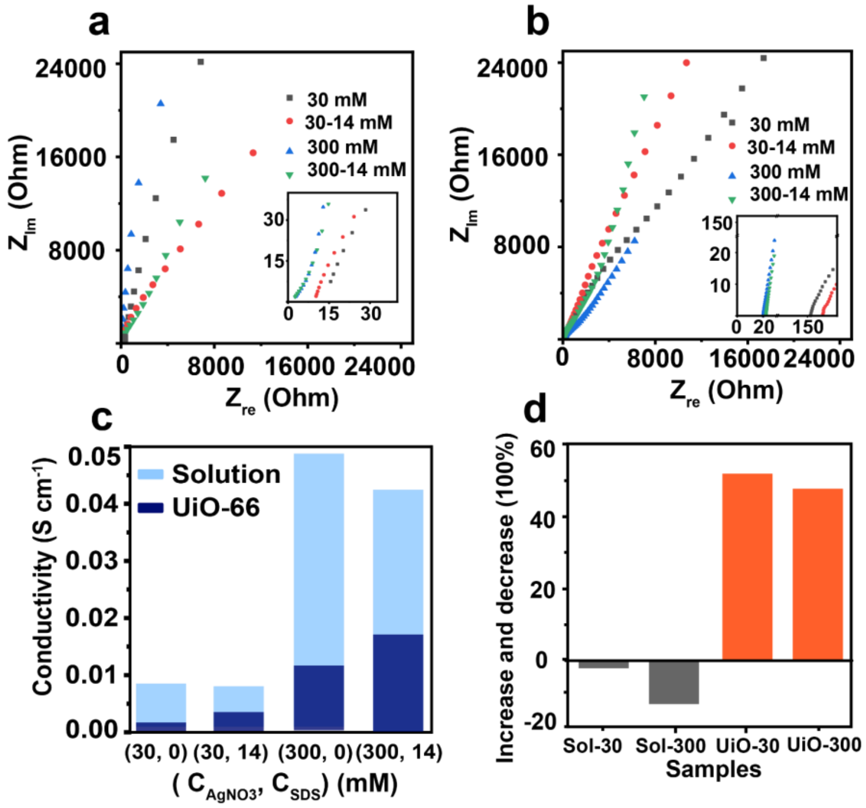

Supplementary Figure 12. Experimentally measured conductivities of the UiO-66 octahedron pellet and the electrolyte solutions. (**a**, **b**) EIS plots of the UiO-66 octahedron pellet and the electrolyte, respectively. (**c**) The calculated conductivities of the UiO-66 pellet and the electrolytes. (**d**) The increase and the decrease of the conductivities of the UiO-66 pellet and the electrolyte after adding 14 mM SDS. Sol-30: Electrolyte composed of 30 mM $AgNO_3$. Sol-300: Electrolyte composed of 300 mM $AgNO_3$. UiO-30: The UiO octahedron pellet filled with the electrolyte composed of 30 mM $AgNO_3$. UiO-300: The UiO octahedron pellet filled with the electrolyte composed of 300 mM $AgNO_3$. Source data are provided as a Source Data file.

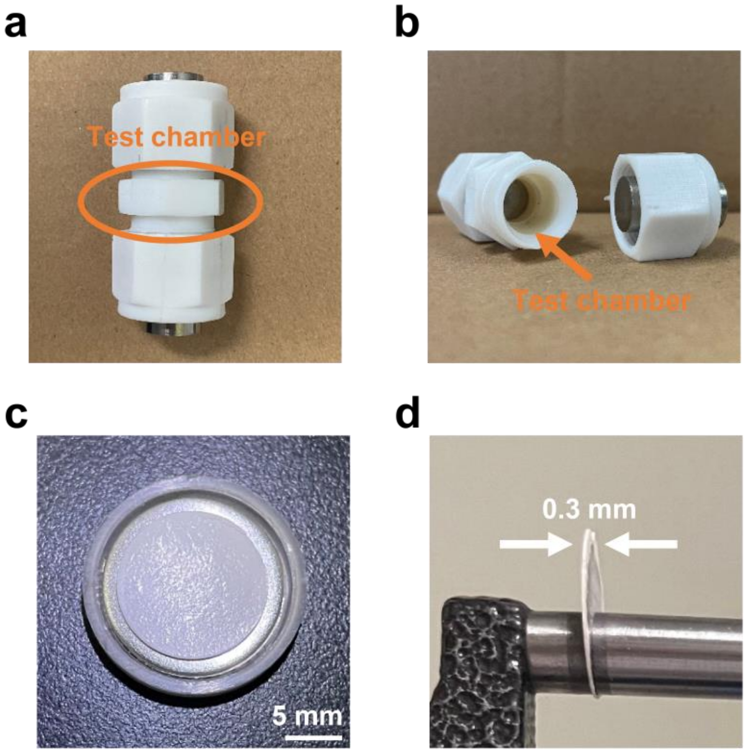

Supplementary Figure 13. Equipment used for the electrochemical measurements. (**a**, **b**) The customized swagelok cell. The diameter and the thickness of the chamber were 0.73 cm and 0.51 cm, respectively. (**c**, **d**) The pressed UiO-66 octahedron pellet for EIS measurements. The diameter and the thickness of the pressed pellet were 0.65 cm and 0.03 cm, respectively.

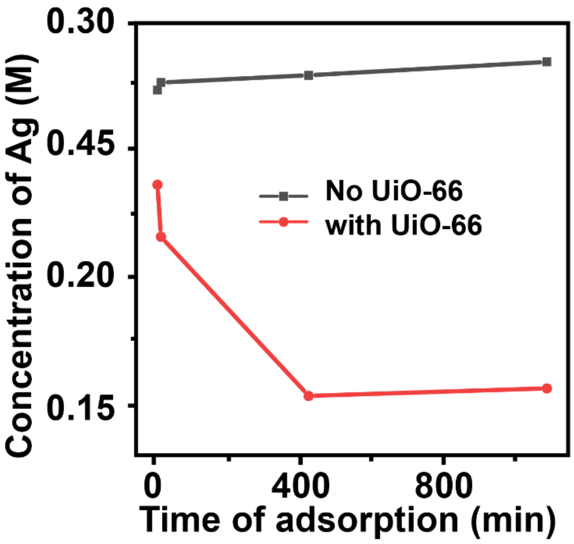

Supplementary Figure 14. The adsorption of Ag^+ ions by the UiO-66 octahedron powders. The remaining concentration of Ag^+ ions in the solution after different adsorption times. Source data are provided as a Source Data file.

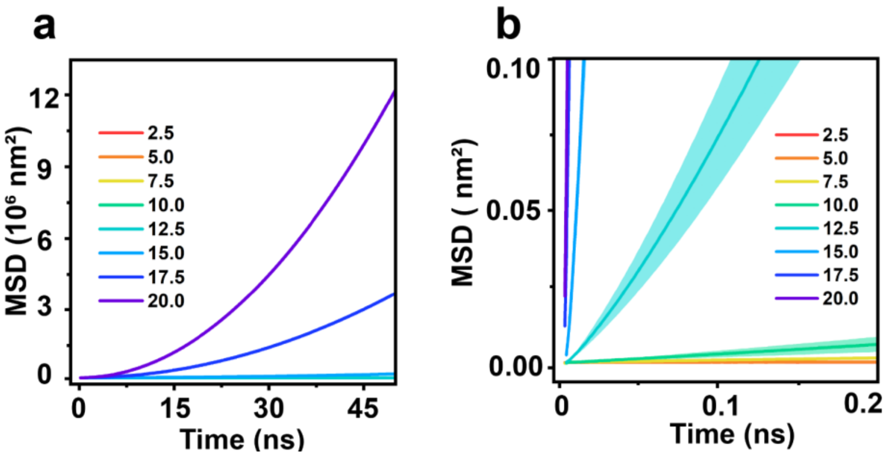

Supplementary Figure 15. The calculated MSD. (**a**) Time evolution of the one-dimension MSD for Ag^+ ions in UiO-66 octahedra when different numbers of Ag^+ ions existed in the unit cell. (**b**) Enlarged observation in the first 0.2 ns. Source data are provided as a Source Data file.

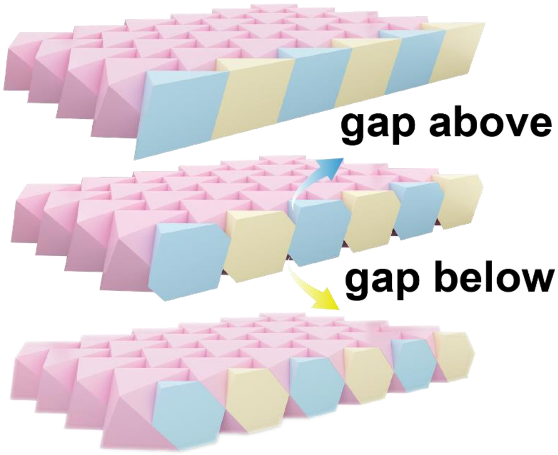

Supplementary Figure 16. Schematics of different sectional views of the monolayer UiO-66 octahedron template. From top to bottom: Cut from the middle of the UiO-66 octahedra, from a random position of the gaps, and from the middle of the gaps, respectively.

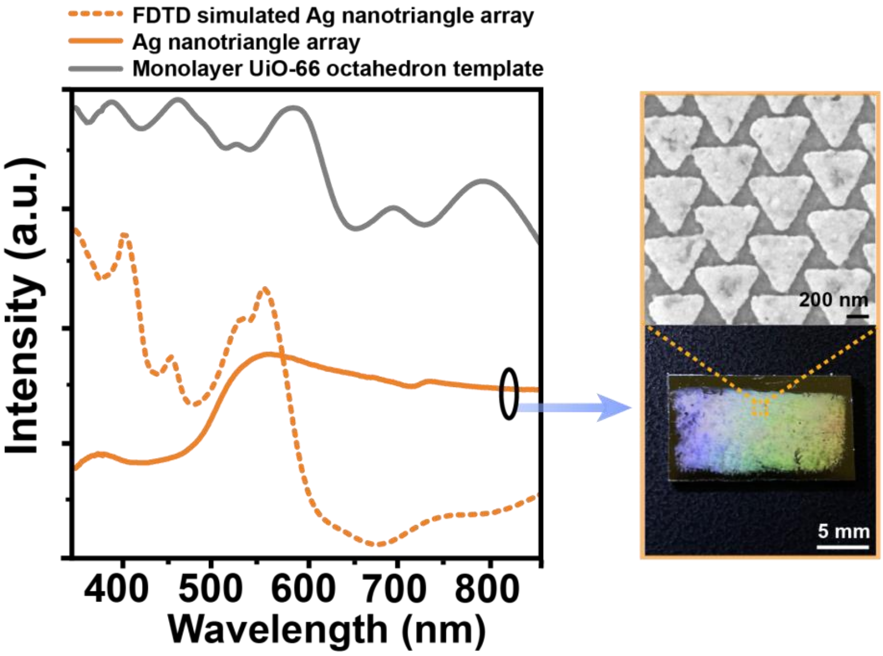

Supplementary Figure 17. The FDTD simulated and experimentally measured extinction spectra. The photo and the SEM image of the Ag nanotriangle array were shown at the right column. The electrodeposition time of the Ag nanotriangle array was 15 s. Source data are provided as a Source Data file.

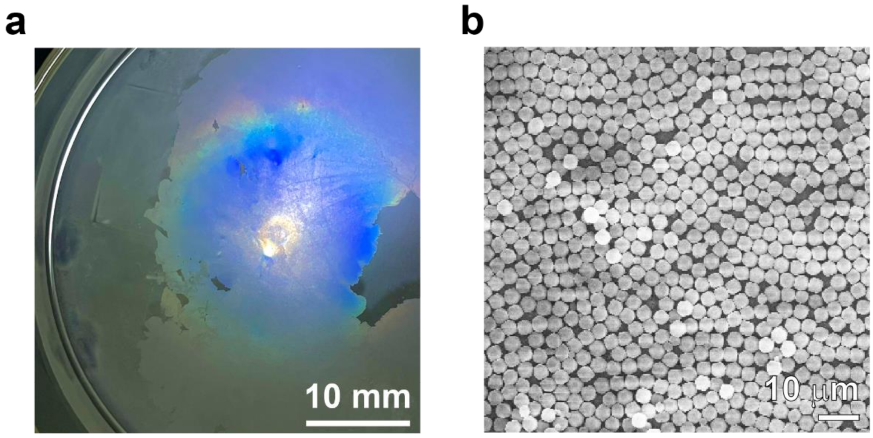

Supplementary Figure 18. The self-assembled monolayer MIL-96 THB template. (**a**) The monolayer MIL-96 THB template floating on the water surface. (**b**) SEM image of the self-assemble MIL-96 THB template.

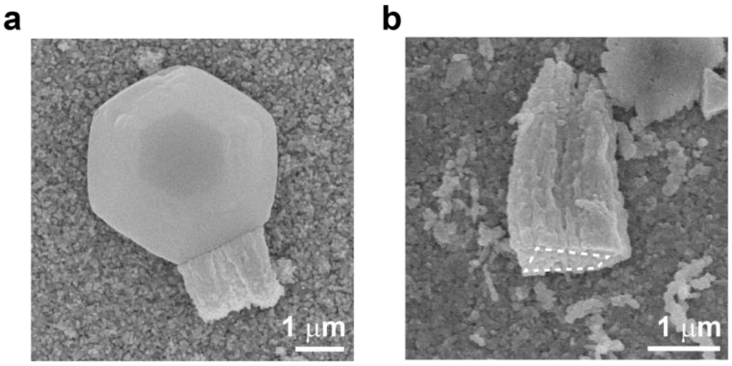

Supplementary Figure 19. Peeling off the MIL-96 microparticle template after Ag electrodeposition. (**a**) An Ag micropillar connected to a facet of a MIL-96 microparticle. (**b**) An Ag micropillar detached from a MIL-96 microparticle. The dotted polygon marked the shape of the micropillar's top surface, which is the same as the shape of the facet of the MIL-96 microparticles.

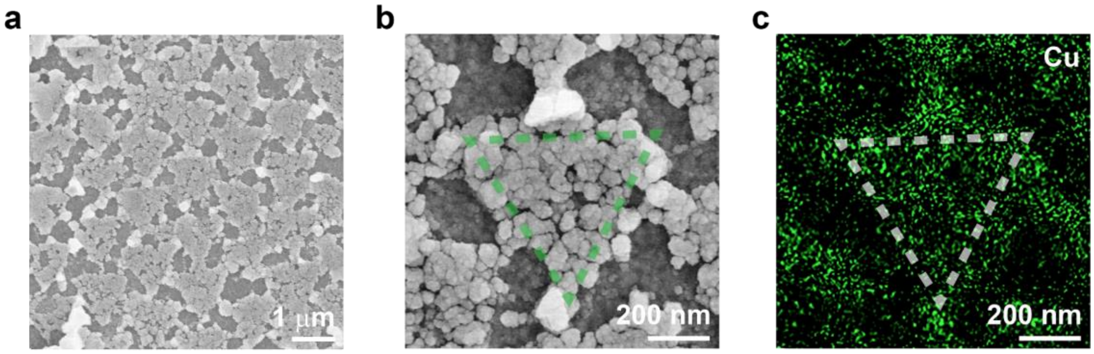

Supplementary Figure 20. SEM images and element mapping of Cu nanotriangle arrays electrodeposited using the monolayer UiO-66 octahedron template. (**a**, **b**) SEM images with different magnifications. (**c**) Element mapping of Cu.

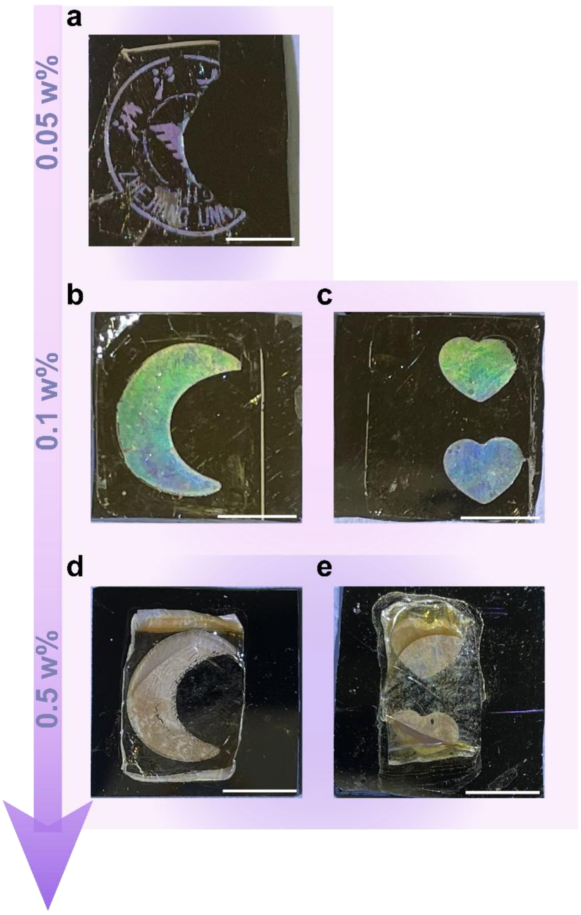

Supplementary Figure 21. UiO-66@PBI composite film with different amounts of PBI. (**a**) The mass concentration of PBI solutions was 0.05 wt.%. (**b**, **c**) The mass concentration of PBI solutions was 0.1 wt.%. (**d**, **e**) The mass concentration of PBI solutions was 0.5 wt.%. The mass concentration was the ratio of the mass of PBI to that of PBI and NMP. Scale bar: 5 mm.

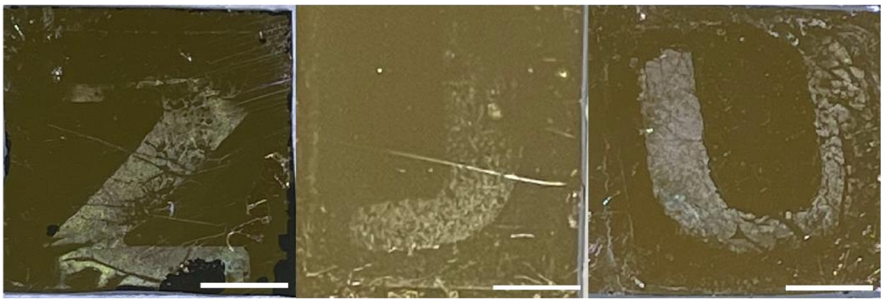

Supplementary Figure 22. Electrodeposited letters using the UiO-66@PBI template. The letters were formed by Ag nanotriangles. The electrodeposition time was 5 s. The electrodeposition voltage was 1.5 V. Scale bar: 5 mm.

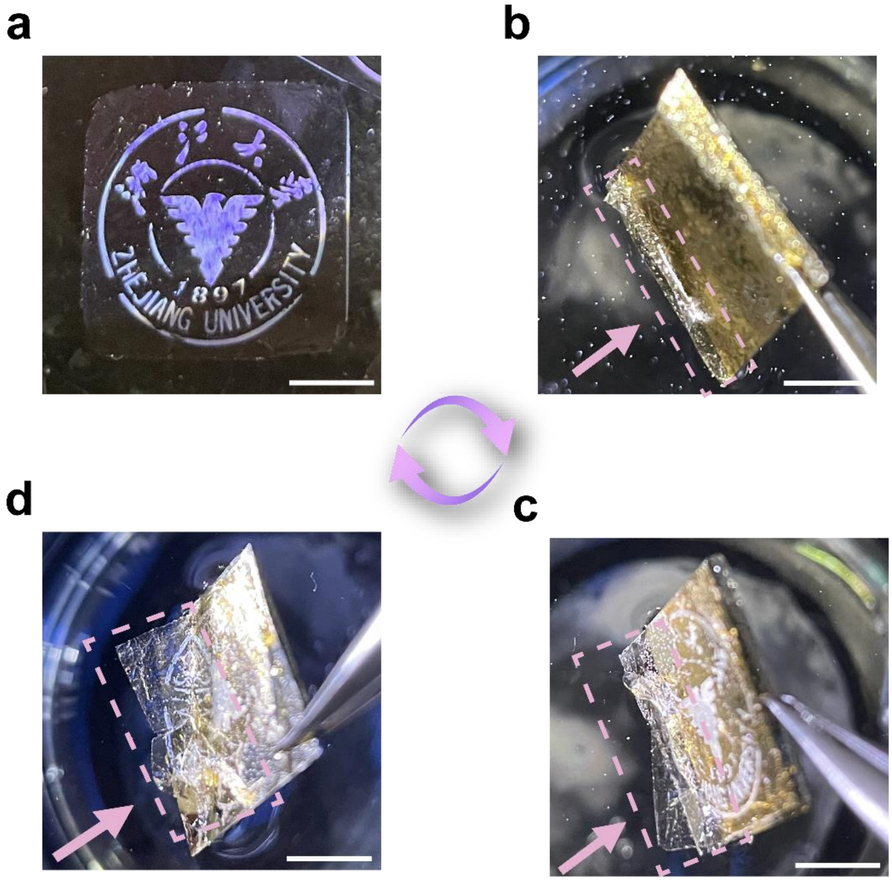

Supplementary Figure 23. Peeling off the UiO-66@PBI template from the electrodeposited Ag nanotriangle array. (**a**) UiO-66@PBI template floating on the water surface. (**b**) Immersing the UiO-66@PBI template after Ag electrodeposition into ethanol and water in sequence. (**c**) The UiO-66@PBI template started to detach from the underneath Ag nanotriangles. (**d**) The UiO-66@PBI template was almost detached from the underneath Ag nanotriangles. Scale bar: 5 mm.
