# Supplementary material for: Metal-organic framework template-guided electrochemical lithography on substrates for SERS sensing applications
Source: Nat Commun. 2023 Sep 20;14:5860. doi: 10.1038/s41467-023-41563-5 (PMC10511444; doi:10.1038/s41467-023-41563-5)
Supplement: Supplementary file 3 — Description of additional supplementary files [file 41467_2023_41563_MOESM3_ESM.pdf]

# Description of Additional Supplementary Files

**Supplementary Movie 1:**

Self-assembly process of the UiO-66 octahedra into densely packed monolayer films.

**Supplementary Movie 2:**

Molecular dynamics simulated transportation process of $Ag^+$ ions within the UiO-66 microparticles in the electrolyte composed of low concentrations of $Ag^+$ ions.

**Supplementary Movie 3:**

Molecular dynamics simulated transportation process of $Ag^+$ ions within the UiO-66 microparticles in the electrolyte composed of high concentrations of $Ag^+$ ions.
